# Brief Review: Racial and Ethnic Disparities in Cardiovascular Care with a Focus on Congenital Heart Disease and Precision Medicine

**DOI:** 10.1007/s11883-023-01093-3

**Published:** 2023-03-25

**Authors:** Joseph Bayne, Jonah Garry, Michelle A. Albert

**Affiliations:** 1 Division of Cardiology, Department of Medicine, University of California San Francisco, 505 Parnassus Ave, San Francisco, CA 94143-0474 USA; 2 Department of Medicine, University of California San Francisco, San Francisco, CA USA; 3 CeNter for the StUdy of AdveRsiTy and CardiovascUlaR DiseasE (NURTURE Center), Division of Cardiology, Department of Medicine, University of California at San Francisco School of Medicine, San Francisco, CA USA

**Keywords:** Racial and ethnic disparities, Precision medicine, Social determinants of health, Cardiovascular disease, Adult congenital heart disease

## Abstract

**Purpose of Review:**

This is a brief review about racial and ethnic disparities in healthcare with focused attention to less frequently covered areas in the literature such as adult congenital heart disease, artificial intelligence, and precision medicine. Although diverse racial and ethnic populations such as Black and Hispanic groups are at an increased risk for CHD and have worse related outcomes, they are woefully underrepresented in large clinical trials. Additionally, although artificial intelligence and its application to precision medicine are touted as a means to individualize cardiovascular treatment and eliminate racial and ethnic bias, serious concerns exist about insufficient and inadequate available information from diverse racial and ethnic groups to facilitate accurate care. This review discusses relevant data to the aforementioned topics and the associated nuances.

**Recent Findings:**

Recent studies have shown that racial and ethnic minorities have increased morbidity and mortality related to congenital heart disease. Artificial intelligence, one of the chief methods used in precision medicine, can exacerbate racial and ethnic bias especially if inappropriate algorithms are utilized from populations that lack racial and ethnic diversity.

**Summary:**

Dedicated resources are needed to engage diverse populations to facilitate participation in clinical and population-based studies to eliminate racial and ethnic healthcare disparities in adult congenital disease and the utilization of artificial intelligence to improve health outcomes in all populations.

## Introduction

Well-described racial and ethnic cardiovascular disease disparities persist despite advances in therapies that have improved cardiovascular morbidity and mortality in the general population [1]. Racial categorizations are social constructs that albeit demonstrate differences in health outcomes based on race as well as ethnicity. However, within all racial/ethnic populations, significant heterogeneity exists based in part on the history of enslavement, culture, and geography. Therefore, within each racial/ethnic group, differences in disease prevalence and life expectancies exist.

Cardiovascular health disparities are largely attributed to differences in the prevalence of conditions such as obesity and hypertension and social determinants of health including but not limited to socioeconomic factors among diverse racial and ethnic groups living in the USA. African American/Black (Black) and Hispanic/LatinX (Hispanic) persons have the most robust cardiovascular disparities data. Among various groups, distinct differences are also observed based on ethnicity. For example, certain Asian populations such as Filipinos and South Asians have more prevalent cardiovascular risk factors and worse cardiovascular outcomes compared to White patients [2, 3].

This brief review will focus on racial/ethnic disparities in congenital heart disease, clinical trials, and precision medicine. Few data are available about congenital heart disease according to race/ethnicity. While precision medicine is celebrated as the future of health care and a means to individualize cardiovascular treatment, concern exists about the availability of sufficient data sources comprised of diverse racial and ethnic populations. Hence, adequate representation of all populations, particularly those who are traditionally disadvantaged, is needed in research studies to help eliminate cardiovascular health care disparities.

## Overview of Disparities in Congenital Heart Disease

### Gestational Risk Factors Associated with Congenital Heart Disease

Most literature about racial and ethnic health disparities in the USA focuses on risk factors in adults. However, emerging data suggests that environmental exposures in utero are associated with increased later cardiovascular disease in adulthood and that diverse racial/ethnic patients are more likely to be at risk for these exposures [4]. For instance, children of mothers with diabetes (type I or type II) during pregnancy are five times more likely to have congenital heart disease (CHD) [5, 6]. Elevated maternal pre-pregnancy body mass index (BMI) is associated with cardiac abnormalities in offspring, especially conotruncal and right ventricular outflow tract defects [7]. Prevalent diabetes during pregnancy is more common among women from racial/ethnic diverse groups particularly among East Asian and South Asian women [8, 9], while rates of obesity and hypertension pre-pregnancy are significantly higher in Black and Hispanic women [10].

Although thus far few genes have been linked to congenital heart defects, maternal congenital heart disease generally carries a 3% to as high as 50% (in single gene defects with autosomal dominant inheritance such as Marfan’s disease) chance of a defect in offspring [11]. Available studies demonstrate different rates of specific congenital defects in White, Hispanic, Black, and Asian persons [12–14]. One study from the UK showed that babies of both Asian and African descent were significantly more likely to have CHD than White babies [15]. Rates of pre-pregnancy diabetes and pre-pregnancy BMI were not recorded. Future research is needed that includes data about known risk factors, as well as maternal race/ethnicity to better understand the association between race/ethnicity and the risk of congenital cardiac defects.

### Congenital Heart Disease in Children and Adults

Evidence indicates that racial and ethnic disparities exist in congenital heart disease outcomes in children as well as among individuals with CHD who survive to adulthood. Racial/ethnically diverse babies with CHD are more likely to have surgical complications compared to White babies born with the same abnormalities. For example, a cohort study involving 1796 patients in CA, USA, looked at the composite outcome of readmissions and mortality within the first year of life for White, Hispanic, Asian, and Black babies born with hypoplastic left heart syndrome and dextro-transposition of the great arteries. Hispanic patients had worse overall outcomes, were more likely to experience care lapses, and had a greater loss to follow-up after diagnosis or an intervention. Although outcomes were not worse among Asian and Black patients compared to White patients, Black patients only accounted for a small proportion of the study population [16]. Another study evaluating 30-day readmission rates for patients aged 18 years and older after adult CHD surgery demonstrated that Black patients had higher readmission rates compared to White and Hispanic patients [17]. Multiple studies show similar findings, and while the exact reasons are uncertain, limited financial resources, outpatient access to care, and provider bias all likely contribute [18, 19].

Death rates are also higher in Black and Hispanic patients born with CHD. A Texas, USA, registry consisting of babies born with CHD between 1996 and 2003 showed higher mortality among Black and Hispanic patients across multiple congenital heart disease subtypes. Similarly, research using death certificates filed in the USA from 1999 to 2006 revealed significantly more deaths among Black patients compared to White and Hispanic patients, especially from birth to 4 years of age. Despite declining mortality each year in all groups, Black patients persistently had higher death rates compared to other racial/ethnic groups [20]. Prospective research is needed that includes larger sample sizes to improve the understanding of the relationship between race/ethnicity and congenital heart disease to help identify targeted preventive strategies. Large clinical trials have traditionally been difficult in CHD patients due to the diversity of cardiac malformations and historically low survivability of CHD patients. However, the survivability of CHD patients has increased dramatically of late with over 97% of children expected to reach adulthood [21], thereby increasing the urgency for the need of large clinical trials that are able to adequately enroll diverse racial and ethnic patients.

#### Racial/Ethnic Disparities in Large Clinical Trials

In fact, cardiovascular care advances for adults in the past 30 years and the associated decrease in morbidity and mortality are attributed to landmark trials demonstrating the efficacy of certain treatments and prevention measures [22]. Besides issues pertaining to enrollment of diverse racial and ethnic groups, the exclusion of entire countries exists due to their lack of significant financial and research resources. Of concern is that persons of Indigenous, African, and Hispanic descent are usually not included regardless of country of residence. For example, in the USA, Black and Hispanic patient representation in clinical trials is extremely poor, having a clinical trial prevalence of 5% and 1%, respectively [23]. Another issue of significance is the lack of main principal investigators from these respective groups [24]. This lack of representation of diverse racial and ethnic populations in clinical trials and other research studies contributes to health disparities within cardiovascular disease through the promotion of group distrust along with a lack of understanding about whether developed interventions will be maximally efficacious.

## Personalized Medicine/Precision Medicine

While large clinical trials brought about revolutionary changes to cardiac care, precision medicine is anticipated to bring about the next generation of advances in health care and has gained more focus and popularity in recent years [25]. Precision medicine is defined as an approach for disease treatment that stratifies patients based on large-scale data that includes lifestyle, clinical, environmental, and molecular information with subsequent tailored treatments for patients based on their individual profiles [26]. In cardiovascular disease, precision medicine offers promise to optimize blood pressure and cholesterol medication based on specific characteristics. Electronic medical records, the internet, and smartphones facilitate data collection about the patient’s environment, behaviors, and ambulatory heart rate and blood pressure trends in real-time. This information can be potentially tailored to optimize patient-specific treatments [27]. Additionally, precision public health might be an approach for addressing sociodemographic disparities in care and outcomes given the relatively large impact of social determinants of health.

Two ongoing national programs, the NIH All of US program and the NHLBI Trans-Omics for Precision Medicine (TOPMED) program, are notable efforts to facilitate diverse enrollment in precision medicine initiatives. The All of Us program was launched in 2018 aiming to enroll 1 million participants with biospecimens, electronic health record data, physical measurements, and questionnaires [28]. The All of Us database is also serving to track data about COVID-19 including health outcomes, social, economic effects, and the significance of SARS-CoV-2 antibodies [29]. Launched in 2014, TOPMED seeks to provide precision management tied to individual genetics and environment through whole-genome sequencing, metabolomics, epigenomics, proteomics, and transcriptomics along with clinical, environmental, behavioral, molecular, and imaging data [30]. TOPMED has already yielded insights into cardiovascular mechanisms of disease within racial and ethnic minority populations, including the association of elevated D-dimer with risk for cardiovascular events and mutations at the F3 gene locus in subpopulations of women of African descent [30, 31].

Cardiovascular disease is largely a polygenic condition with genetics representing less than 25% of cardiovascular disease and behavioral, social, and environmental factors accounting for most of the condition. Notwithstanding the importance of other influences, genetic illnesses are understudied in diverse racial/ethnic populations. Historically, genetics research has focused on single-gene disorders with extreme phenotypes such as cystic fibrosis. However, single-gene disorders affecting Black individuals such as sickle-cell anemia were largely understudied and still receive substantially less research funding than other conditions despite having high prevalence and high mortality [32–34].

As the understanding and ease of genetic analysis continue to improve, the field of genetics has shifted from single-gene disorders to large-scale multifactorial polygenetic diseases. Genome-wide association studies that utilize large databases of multiple allelic variants to assess disease phenotypes have a paucity of diverse racial/ethnic groups, with persons of African or Latin American and Native/Indigenous ancestry persons comprising 2% and 4% of datasets, respectively [35]. This can lead to the misclassification of genetic diseases in these populations with serious potential consequences such as unnecessary anxiety and treatments. In fact, it was found that African Americans who had benign variant alleles were much more likely to be given an incorrect classification of “pathogenic” due to the low representation of patients with similar ancestry in available exome databases. These misclassifications were corrected when databases were updated with more diverse cohorts [36].

Precision medicine also relies on artificial intelligence to interpret data and apply knowledge to individuals. Artificial intelligence “learns” by applying algorithms to annotated datasets, an approach that can contain biases that apply to sex, culture, and race/ethnicity if the source dataset is not sufficiently diverse [37]. Machine learning programs improve predictive accuracy by optimizing programming for individuals or variables that appear more frequently. In medical databases where racial/ethnic diversity is lacking, algorithms specifying “normal” vs. “abnormal” can potentially be harmful to patient assessment and treatment [38].

## Key Drivers of Health Disparities and Examples of Interventions

As noted, health disparities are tied to structural economic and social inequalities. Life expectancy and multiple health factors closely associated with cardiovascular outcomes and mortality are linked to access to quality education, neighborhood environment, and economic opportunity. Race and ethnicity are major predictors of wealth, particularly intergenerational wealth, and wealth is closely tied to social factors that either promote or derail health risks. In fact, the median incomes of US White and Asian households are $65,900 and $87,200, respectively, whereas those of Hispanic and African American households are $43,800 and $51,400, respectively [39–41].

Psychosocial stress is also associated with cardiovascular risk, and persons belonging to racial/ethnic diverse groups are more likely to have chronic psychosocial stressors and experience depression and anxiety symptoms throughout their life. Some work indicates that reported cumulative psychosocial stress is higher in Black women compared to Hispanic, Asian, and White women [42]. Psychosocial stress among African American women is also tied to higher cardiovascular disease risk [43, 44].

Housing is also linked to health outcomes. Historically, Black persons living in the USA have been prevented from accessing quality housing in desirable neighborhoods. Lack of quality housing is related to increased rates of depression and anxiety, as well as increased exposure to pollutants and chemicals such as asbestos leading to respiratory and cardiovascular diseases. For example, in a systematic review of multiple housing intervention studies from 1887 to 2007, it was shown that improvement of housing conditions through the provision of insulation and heating consistently improves overall health outcomes [45]. Black and Hispanic individuals are more likely to live in food deserts with poor access to affordable fresh produce with consequently increased prevalence of obesity, hypertension, diabetes, and heart disease [46]. Access to fruits and vegetables can be improved through multifaceted health initiatives such as those implemented in New York City since 2002 [47]. Increases in the availability of fruits and vegetables in schools and the provision of “health bucks” or food finance supplements/stamps for utilization at farmer’s markets and green carts significantly increased daily fruit and vegetable consumption [47].

System-wide interventions are challenging to implement and often require a substantial allocation of resources, but they effectively improve population health. Expanding health coverage is often proposed as a more targeted health intervention to improve health outcomes for many people and potentially address racial health disparities. For example, Medicaid expansion under the Affordable Care Act increased insurance coverage and was associated with decreasing disparities in Black and Hispanic persons, including improved heart transplantation health outcomes among Black individuals [48, 49•]. Interestingly, however, although Massachusetts health reform increased insurance coverage to many previously uninsured people including Blacks, Hispanics, and Asians, it did not increase the number of coronary revascularization procedures performed in Black and Hispanic patients, a finding similar to observed data from the Veterans Affairs Health System [50•]. This suggests that equalizing access to care and narrowing economic gaps alone will not eliminate health disparities.

Differences in health outcomes after control for co-morbidities and provision of equitable health insurance options likely result from subconscious implicit biases. For example, in a study examining 1.8 million births in Florida between 1992 and 2015, Black newborns were 50% less likely to die if their provider was a Black physician [51••]. This sobering statistic is extremely relevant as only 5% and 6% of US physicians are Black and Hispanic persons, respectively [52]. Increasing the number of healthcare providers in these racial/ethnic groups is one solution that could help address racial disparities. The implicit association test is one metric utilized to assess implicit racial bias [53]. However, research is needed to determine which methods of training are most effective in reducing implicit biases and their contribution to discrepancies in provider care.

Engaging patients where they gather in community settings is a promising means of addressing some healthcare disparities. For example, barbershops can be utilized as a locus of care. Compelling evidence for this approach comes from a cluster-randomized trial for hypertension management in African American men. Patients were randomized to barbers-encouraging meetings at barbershops with pharmacists who helped prescribe medications compared to receipt of encouragement to attend medical appointments and take medications. Encouragement partnered with a pharmacist’s intervention at barbershops had a marked increase in medication compliance and blood pressure control compared to encouragement alone [54]. This intervention addressed individual factors of trust in the healthcare system and provider biases, as well as systems-level factors including access to quality healthcare and community engagement. Importantly, it also brought healthcare directly to the community. Culturally competent and congruent strategies also receive success in other racial and ethnic groups and other fields of medicine especially when implemented across multiple levels of health care. For example, outcomes for Latino children in the Pediatric Intensive Critical Care Unit greatly improved when hospital staff who spoke Spanish were hired, healthcare providers were educated in culturally competent care, and outreach efforts that removed barriers to healthcare access were implemented [55].

## Conclusion

**Fig. 1:**
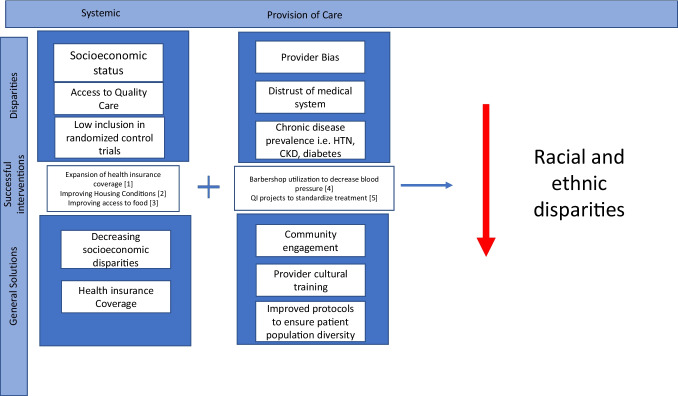
Successful solutions to racial and ethnic disparities at the systemic and provider levels. This figure is organized into 3 rows and 2 columns. The rows are divided into categories titled “Disparities,” “Successful Interventions,” and “General Solutions,” and the columns further organize each of these into categories at the systemic level and the provision of care level. The figure shows that interventions at both the systemic and provider level are needed to effectively address racial disparities in cardiovascular care. (From [45, 47, 48, 54, 56])

The reasons for racial and ethnic disparities in cardiovascular disease are multifactorial including lack of representation of main principal investigators from diverse racial/ethnic backgrounds and diverse patients in clinical studies, as well as from socioeconomic disparities couched in discriminatory practices over time. As new treatments and technologies develop that are geared towards treating individual patient’s needs, inclusiveness is imperative to ensure equity. Novel treatments and technology alone will not eliminate racial and ethnic health disparities. Ongoing efforts to understand contributing factors to racial/ethnic healthcare disparities and the application of evidence-based methods to address them will promote better health outcomes for all communities. Successful solutions must occur at multiple levels including provider and systems approaches (Fig. 1).
